# Supplementary material for: Integrated analysis of the aqueous humor microbiome and lens capsule transcriptome in high myopia cataract: a pilot study
Source: Front Med (Lausanne). 2026 Jun 16;13:1845205. doi: 10.3389/fmed.2026.1845205 (PMC13314463; doi:10.3389/fmed.2026.1845205)
Supplement: Supplementary file 3 [file Data_Sheet_1.zip › 6.PICRUSt2/Meta_Statistic/C372089/Wilcoxon/COG_abundance.Wilcoxon.pdf]

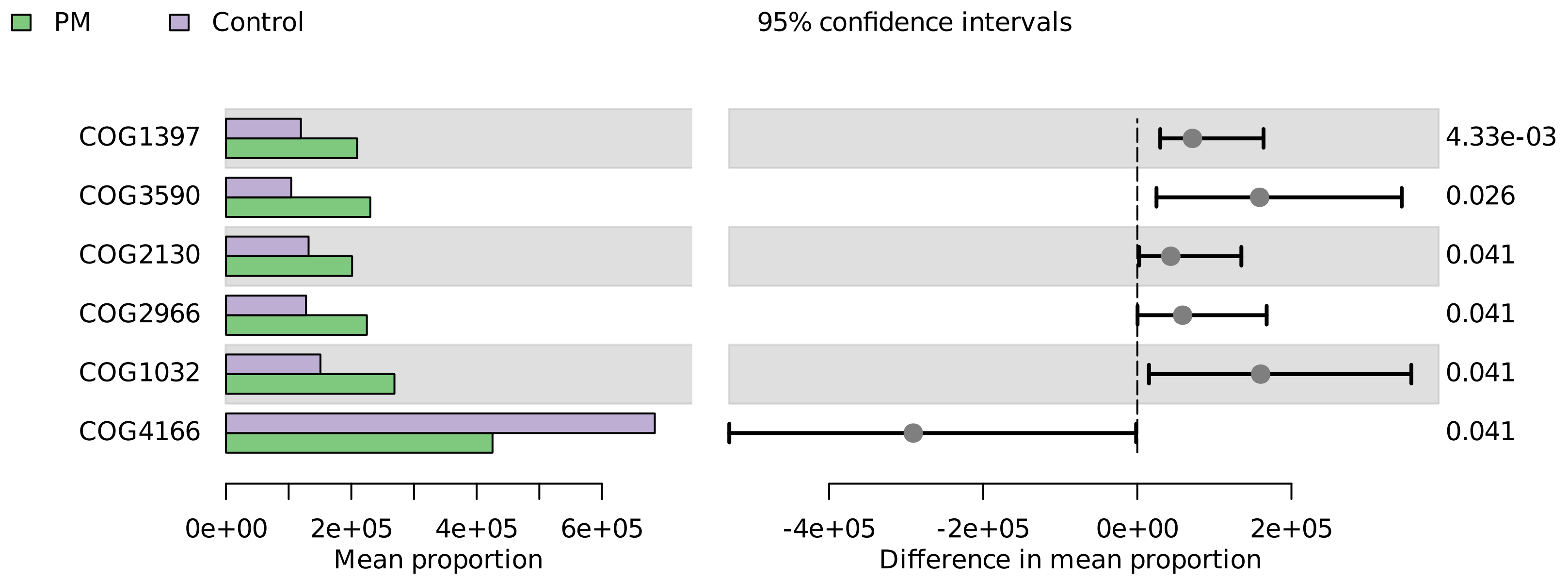

PM
Control
95% confidence intervals
COG1397
COG3590
COG2130
COG2966
COG1032
COG4166
4.33e-03
0.026
0.041
0.041
0.041
0.041
0e+00
2e+05
4e+05
6e+05
Mean proportion
-4e+05
-2e+05
0e+00
2e+05
Difference in mean proportion
